# Insights into the Tumor Microenvironment—Components, Functions and Therapeutics

**DOI:** 10.3390/ijms242417536

**Published:** 2023-12-15

**Authors:** Kornélia Baghy, Andrea Ladányi, Andrea Reszegi, Ilona Kovalszky

**Affiliations:** 1 Department of Pathology and Experimental Cancer Research, Semmelweis University, 1085 Budapest, Hungary; kovalszky.ilona@med.semmelweis-univ.hu; 2 Department of Surgical and Molecular Pathology and the National Tumor Biology Laboratory, National Institute of Oncology, 1122 Budapest, Hungary; ladanyi.andrea@oncol.hu; 3 Department of Pediatrics, College of Medicine, University of Florida, Gainesville, FL 32610, USA; reszegi.andrea.margit@semmelweis.hu; 4 Department of Pathology, Forensic and Insurance Medicine, Semmelweis University, 1091 Budapest, Hungary

**Keywords:** tumor microenvironment, extracellular matrix, proteoglycans, tumor-associated fibroblasts, tumor immunity, immune checkpoint inhibitors, tumor stroma, angiogenesis

## Abstract

Similarly to our healthy organs, the tumor tissue also constitutes an ecosystem. This implies that stromal cells acquire an altered phenotype in tandem with tumor cells, thereby promoting tumor survival. Cancer cells are fueled by abnormal blood vessels, allowing them to develop and proliferate. Tumor-associated fibroblasts adapt their cytokine and chemokine production to the needs of tumor cells and alter the peritumoral stroma by generating more collagen, thereby stiffening the matrix; these processes promote epithelial–mesenchymal transition and tumor cell invasion. Chronic inflammation and the mobilization of pro-tumorigenic inflammatory cells further facilitate tumor expansion. All of these events can impede the effective administration of tumor treatment; so, the successful inhibition of tumorous matrix remodeling could further enhance the success of antitumor therapy. Over the last decade, significant progress has been made with the introduction of novel immunotherapy that targets the inhibitory mechanisms of T cell activation. However, extensive research is also being conducted on the stromal components and other cell types of the tumor microenvironment (TME) that may serve as potential therapeutic targets.

## 1. Introduction

The treatment of cancer has made tremendous strides in the past decade. Despite this, there are numerous instances of curative intention failing in the battle between the tumor and the patient’s body; the tumor will prevail, and the patient will perish. The statistical evidence that the number of malignant diseases increases annually is also thought-provoking [1]. When treating malignancies, the question arises as to whether it is sufficient to target only the tumor cells with therapy. Like our healthy organs and tissues, the tumor tissue is an ecosystem and employs solutions detrimental to health to ensure its survival. Therefore, we must consider the tumor components that were formerly regarded as “innocent” and that contribute to the survival of cancer cells. Cancer is a heterogeneous disease characterized by the uncontrolled growth and proliferation of abnormal cells. Yet, accumulated evidence has disclosed the vital role of the tumor microenvironment (TME) in cancer growth control. The TME is a complex system comprising three main components: (1) cellular components, such as stromal cells, fibroblasts, immune cells and endothelial cells, the function of which changes as a result of their interaction with the tumor cells; (2) extracellular components, which are in part the proteins that compose the extracellular matrix (ECM); (3) soluble components, which collectively shape tumor initiation, progression and therapeutic response. The interactions between cancer cells and the constituents of the TME influence tumor growth, immune escape, angiogenesis and metastasis formation [2,3,4]. In the present review, we aimed to provide a concise overview of the major acellular and cellular components of the extracellular matrix and discuss the role of the immune system in modulating the tumorous microenvironment as well as the key features of tumor angiogenesis. Lastly, we provide insight into therapeutic approaches targeting components of the tumorous stroma.

## 2. Extracellular Matrix Components and Their Functions

**Figure 1:**
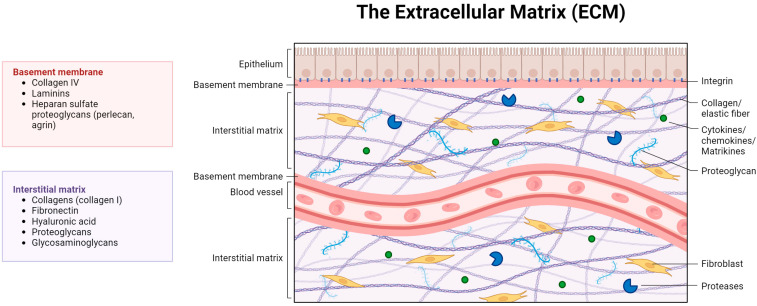
Schematic illustration depicting the structure and components of the extracellular matrix (ECM). The ECM represents a complex network of proteins that not only forms a support structure for resident cells but also interacts closely with them, modulating their phenotypes and functions.

The ECM is a mesh network of diverse proteins and carbohydrates that surrounds the cells, provides mechanical support and participates in intercellular adhesions and communication and in cell migration (Figure 1).

### 2.1. Collagens

The principal ECM structural proteins are collagens, the components of the fibrous interstitial matrix. Collagen I and collagen III, 2 of the more than 28 known types, are the primary structural components of both the healthy and the tumorous stroma. Long before their oncogenicity was recognized, it was discovered that an increase in their quantity causes rigidity in the tumor matrix. During ECM remodeling, the collagen content of the ECM increases, resulting in ECM stiffness and an unfavorable prognosis. After forming triple-helix structures, collagens undergo several modifications before being secreted into the ECM [5]. In tumor-associated stroma, the original ECM will be replaced as a result of the catalytic function of the matrix metalloproteases MMP-1, -8, -13 and -14 [6,7]. These new collagens are the main stimulators of DDR1 tyrosine kinase receptors on the surface of tumor cells, facilitating tumor cell proliferation [8,9]. 

### 2.2. Adhesive Glycoproteins

#### 2.2.1. Fibronectin

Fibronectin is the principal adhesive glycoprotein in the extracellular matrix. Via integrins, it is linked to epithelial cells. It took a long time for its oncogenicity to become evident, and there are still contradictory data available. Fibronectin is produced by fibroblasts and by other tumor-derived stromal and cancer cells. It is the permanent component of a fibrous ECM [10]. In addition to its myriad physiological functions, a growing body of evidence supports the role of fibronectin in the biological behavior of tumors [11]. In malignancies, fibroblasts and macrophages are primarily responsible for fibronectin production. Fibronectin influences tumor cell migration and invasion, as well as tumor angiogenesis [12].

#### 2.2.2. Laminins

Laminins are the structural components of the basement membrane. There are 15 known different types of laminins. Their structure consists of three chains (alpha, beta and gamma). Laminin-5 is one of the primary participants involved in the formation of the dermal–epidermal connection, also known as hemidesmosome. Beside its normal functions, a number of publications reported the involvement of laminin 5 in tumor progression [13]. Its interaction with collagen VII facilitates the development of skin cancer. Ensuring the stability of laminin in the basement membrane is required for the support of the epithelial layer [14]. It is linked to epithelial cells through integrin molecules (α6β1, α6β4). Due to the catalytic function of matrix metalloproteases, laminin loses its adhesion properties and penetrates the interstitial matrix of the tumor stroma, mainly via its gamma chain, where it promotes the invasion of tumor cells [15,16]. Tumor-associated fibroblasts stimulate the progression of cervical cancer by elevating laminin-1 expression in the tumor stroma [17]. Recently, the LAMC2–NR6A1 fusion gene was detected in ovarian cancer, which facilitated tumor growth in experimental models [18]. The number of studies documenting the oncogenic potential of this ECM-associated protein family is continuously rising, indicating its ability to promote tumor growth. 

### 2.3. Proteoglycans

Proteoglycans are composed of a protein chain and glycosaminoglycans that are linked to it via the Ser-Gly amino acid motif [19]. They are found in the ECM, on the surface of epithelial cells and, occasionally, in their cytoplasm and nuclei. In the extracellular matrix, proteoglycans are responsible for stromal turgor and can bind numerous cytokines and growth factors via their sugar chains. In addition to their numerous physiological functions, they may also contribute to the biological behavior of tumors. Based on their structure, proteoglycans can either stimulate or inhibit tumor growth [20,21,22]. The most well-known proteoglycan with antitumor properties is decorin, which inhibits the activity of multiple cell surface tyrosine kinase receptors and of TGF-β [23]. Another, clinically important proteoglycan is glypican-3, a cell surface heparan sulfate proteoglycan, one of the stimulators of liver cancer development and a promising marker in hepatocellular carcinoma [24]. Agrin, localized in the basement membrane, also promotes the formation of liver cancer and is primarily associated with the YAP-TAZ pathway involved in the regulation of matrix rigidity [25]. In the last few years, one proteoglycan, namely, SPOCK1/testican-1 has been gaining more and more attention due to its tumor-promoting effects in cancer. SPOCK1/testican-1 is a heparan sulfate proteoglycan that is present in the cytoplasm of numerous epithelial malignancies. It promotes cancer development in part by activating cell surface and intracellular tyrosine kinase receptors and by increasing DNA synthesis [26].

A major family of heparan sulfate proteoglycans is that of syndecans. The role of syndecans is contradictory. Syndecan-1 is the major cell surface proteoglycan in epithelial cells, a co-receptor in tyrosine kinase signaling. Consequently, it serves a crucial role in the regulation of cellular functions. It is the main proteoglycan in the liver, implicated not only in signaling but also in normal liver functions including lipid metabolism [27]. Whereas it protects against liver cancer in animal studies, it facilitates the development of hepatocellular carcinoma as a receptor of the hepatitis C virus [28]. There are tumors in which syndecan-1 protects against cancer [29,30] and others, such as myeloma and mammary and lung cancer, in which it promotes tumor progression [31]. Considering the nature of syndecan-1, however, one must take into account that it can shed into the ECM, be reabsorbed by tumor cells or enter the nucleus. Consequently, each of these events can influence the actual activity of this proteoglycan [32,33,34,35].

Hyaluronic acid (HA) is the only molecule that forms a linear non-sulfated glycosaminoglycan chain composed of disaccharide units and does not bind to proteins [36]. Depending on its molecular size, HA effects vary significantly. While its large chains serve a crucial role in maintaining connective tissue turgor, inhibiting inflammation and promoting wound repair, low-molecular-weight HA variants bind to the CD44 receptor or to Rham and promote tumor development via the RAS-Raf signal pathway [37]. 

### 2.4. Integrins

Integrins, being heterodimeric cell surface receptors, represent a major category of cell adhesion molecules [38]. In addition to transmitting mechanical forces, the task of integrins is to detect and transmit signals between the cytoskeleton and the ECM. They also play crucial roles in the communication between various cell types and the ECM in the TME [39].

The combination of 18 α- and 8 β-subunits results in a minimum of 24 unique integrin heterodimers. The arsenal of integrins that a particular cell displays determines its ability to adhere to and migrate across various matrices [40].

Particular integrin heterodimers selectively bind to distinct ECM components, such as fibronectin, laminins, collagens, thrombospondin and numerous other adhesion molecules. For instance, αv integrins and integrin α5β1 bind to ligands that contain the RGD sequence [41]. The signals they transmit to cells influence several cell functions. Additional adhesive sequences found in ECM proteins have been identified. These include the EILDV and REDV sequences in spliced fibronectin, which were identified as binding partners of integrin α4β1. Upon attachment to the ECM, integrins assemble in the membrane and attract a variety of adaptor and signaling proteins to form focal adhesions [42]. Despite lacking kinase activity, integrins recruit and activate kinases, including focal adhesion kinases (FAKs) and Src family kinases (SFKs), as well as scaffolding molecules like p130 CRK-associated substrate (p130CAS, also known as BCAR1). Integrins also connect the ECM to the actin cytoskeleton via several proteins, such as vinculin, talin, paxillin, α-actinin and tensin [1]. 

Research examining the function of integrins in the context of the interactions between tumor cells and the so-called cancer-associated fibroblasts (CAFs; see Section 3.1) can take two forms: it can involve examining integrins displayed on CAFs or analyzing integrins on tumor cells that influence CAF functionality or are influenced by CAFs. For instance, the tissue distribution of integrin α10β1 is extremely restricted, as this integrin appears only on chondrocytes and mesenchymal stem cells. However, its upregulation was observed on glioblastoma cells, representing a novel target for this particular type of tumor [43]. α11β1 is a collagen-binding integrin primarily found in subgroups of fibroblasts, with a restricted distribution within tissues [44]. It facilitates the communication of collagens with fibroblasts, promotes their transformation to CAFs and facilitates the development of matrix stiffness [45,46]. In addition, it modulates LOXL1 in non-small cell lung cancer and is expressed on CAFs of various solid tumor types [47]. On the contrary, α3β1, a laminin-binding integrin, is widely distributed across numerous cell types, in both normal and malignant tissues. It appears that this integrin serves not only as a marker of CAFs but also as an essential catalyst for fibroblast differentiation into CAFs within the TME, as seen in pancreas adenocarcinomas [48]. Laminin-332, an ECM protein expressed in the tumor stroma, functions as a ligand for α3β1 integrin and facilitates the transition of tumor-supporting fibroblasts mediated by α3β1 integrin. In contrast, αvβ5 integrins are found on tumor cells where they support tumor cell proliferation and invasion but are also present on endothelial and other stromal cells [49]. αvβ6 integrin is only expressed in epithelial cells, and its main ligand is fibronectin. In contrast to what observed in normal epithelium, its expression increases in epithelial tumors such as colorectal cancer [50], where it participates in the events of epithelial–mesenchymal transformation (EMT). In addition, αvβ6-expressing tumor cells activate resident fibroblasts turning them into CAFs. As a mechanism of action, tumor cells secrete latent TGF-β which is activated by αvβ6 integrin, resulting in fibroblast activation. In turn, CAFs secrete stromal-derived factor-1 (SDF-1), promoting colorectal carcinoma (CRC) metastasis formation. CRC cells and CAFs collaborate to advance the progression of cancer, with integrin αvβ6 contributing to the reciprocal regulation of these cells [51].

### 2.5. Cytokines, Chemokines, Growth Factors and Matrikines

The extracellular matrix contains numerous regulatory molecules, a detailed description of which is beyond the scope of this article. Both cytokines and chemokines such as interleukins, interferons, tumor necrosis factors, etc., play crucial roles in tumor invasion and angiogenesis [52,53,54]. Growth factors (EGF, HGF, PDGF, FGF, TGF-β, etc.) are also important participants, as they transmit signals inside cells by binding to tyrosine kinase and other receptors on the cell surface. Their elevated activity may facilitate pathological signaling in tumors [55,56]. Another group of molecules with recently understood importance are matrikines, cleavage products of matrix proteins [57]. Matrikines can exhibit well-definied biological effects, disinguished from those of their parent molecules. Specifically, these molecules are involved in the regulation of angiogenesis. For example, angiogenesis is stimulated by the perlecan proteoglycan. In contrast, endorepellin, the product of perlecan’s cleavage, inhibits it [58]. Both angiostatin, derived from plasminogen, and endostatin, cleaved from collagen XVIII, inhibit angiogenesis [59]. Versican and its cleavage product versikine play a role in inherited and acquired immune responses. The former interferes with the function of T cells and dendritic cells, whereas the latter promotes the migration of Batf3 dendritic cells and the formation of an immune milieu [60].

### 2.6. Proteases

Uncontrolled proteolysis, elevated protease expression or improper protease activation can all contribute to the development or progression of diseases, including cancer. These enzymes play a role in nearly all aspects of tissue function, with an appropriate share of functions [61]. For a long time, proteases were attributed a role only in tumor invasion, that is, in allowing tumor cell penetration of the limiting basement membrane and in creating an extracellular matrix that would support the formation of metastases. Currently, it is evident that they participate in carcinogenesis, as well as cell division, apoptosis, autophagy and inflammation processes [61,62]. Proteases play a crucial role in promoting EMT, in which the morphology of tumor cells changes, losing epithelial cell characteristics and acquiring fibroblast-like features, thus favoring tumor cell migration and metastasis. Proteases also facilitate tumor cell migration by degrading the basement membrane and extracellular matrix [63]. More than 500 proteases have been identified in the human body, which can be categorized into five groups: metallo-, serine, cysteine, aspartase, and threonine proteases [64,65,66]. 

#### 2.6.1. Metalloproteases

Metalloproteases are essential for pericellular proteolysis and directly affect ECM structure, function and signaling [67,68,69]. Matrix metalloproteases (MMPs), a disintegrin and metalloproteases (ADAMs) and a disintegrin and metalloproteinase with thrombospondin motifs (ADAMTSs) are the three most important and active metalloproteases in the TME. Metalloprotease activity is tightly regulated by TIMPs (tissue inhibitors of metalloproteinases) [70]. Any disturbance of this balance unleashes the proteases’ degradative potency, with MMPs having particularly detrimental effects in cancer [71]. MMPs are classified as collagenases, gelatinases, stromelysins and matrilysins, based on their effect on ECM proteins [68,72,73]. ADAMs usually target the extracellular domains of transmembrane proteins and thus contribute substantially to the cleavage of cell adhesion molecules (e.g., E-cadherin, CD44) [74,75], the shedding of cell surface receptors, the maturation of cytokines and chemokines [76,77,78] and the activation of growth factors [79]. The breakdown of structural ECM proteins is mostly due to ADAMTSs [80]. Hyalectanases target various proteoglycans, others process collagen N-terminal propeptides, and some of them have more specific functions [81,82,83].

#### 2.6.2. Serine Proteases

Trypsin and trypsin-like serine proteases (such as thrombin or tissue factor) have essential functions in the regulation of metabolism, coagulation and blood pressure and have also been linked to cancer, especially in the presence of hemostatic dysregulation [84]. Multiple serine proteases are active players in the immune response. Granzyme B, for instance, is required for apoptosis, which is followed by the clearance of dead cancer cells by the immune system [85]. Neutrophil elastase (ELANE), released into the TME by immune cells, contributes to the remodeling of the ECM [86], the release of growth factors [87] and Toll-like receptor activation [88]. Cathepsin G is implicated in MMP-9 activation, compromises cell adhesion via E-cadherins and enhances TGF-β signaling, thus promoting tumor cell migration [89,90,91,92]. Kallikreins, DPPIV (dipeptidyl peptidase IV), FAP (fibroblast activation protein) and PEP (prolyl endopeptidase) serine proteases are all crucial players in cancer, emerging as clinical markers and prospective diagnostic targets [61].

#### 2.6.3. Cysteine Proteases

Cathepsins cleave the structural proteins of the ECM (collagens, elastins, laminins, glycosaminoglycans, proteoglycans), cell adhesion molecules and cell surface receptors (such as the EGF receptor) [61], affecting signaling pathways involved in cell growth, proliferation and cell death, and fuel the protease pool that drives chronic inflammation [93,94]. Caspases are categorized as inflammatory (caspase-1, -4, -5 and -12 in humans) and apoptotic caspases and are essential for facilitating programmed cell death [95]. Cancer cell resistance to apoptosis is an important hallmark of oncologic transformation [96]. In addition, caspase dysregulation contributes to cancer resistance towards therapeutic intervention [97]. Calpains are calcium-activated proteases with important functions in ECM remodeling, apoptosis regulation and diverse cell signaling pathways [98,99].

#### 2.6.4. Aspartate Proteases

The main cancer-associated aspartic proteases are renin, cathepsins, pepsin C, and napsin A. In addition to hypertension, disturbances of the renin–angiotensin system are linked to pathways deregulated in the pre-cancerous stage [100,101] and can influence immunosuppression in tumors [102]. The cathepsin D lysosomal protease is involved in protein degradation and implicated in tumor progression, angiogenesis and apoptosis [103]. Cathepsin E, an intracellular protease primarily expressed by immune cells, is essential for antigen processing/presentation, apoptosis, cytokine turnover and the regulation of the adipose tissue [104,105]. Significant alterations in pepsin C expression levels have been observed in cancers [106]; however, its diagnostic applications are extremely limited at present. Napsin A, another aspartic protease similar to pepsin, is essential for processing surfactant B in the lungs [107] and is a well-established biomarker for lung adenocarcinoma [108].

#### 2.6.5. Threonine Proteases

Proteasomes are the most important threonine proteases involved in tumorigenesis. They effectively and non-selectively destroy most cellular proteins designated for degradation by the ubiquitin conjugation system [109]. Because of their pivotal role in regulating cell homeostasis, proteasome inhibition is a key strategy in cancer therapy [110,111].

## 3. Cellular Elements of the Tumor Microenvironment

The cellular components of the TME form a dynamically evolving, complex network of cells interacting with each other and with ECM components. The main constituents of the TME are tumor cells, fibroblasts, a variety of immune cell types and cells of the vascular system.

### 3.1. Cancer-Associated Fibroblasts (CAFs)

Cancer-associated fibroblasts are dominant residents of the tumor stroma. Regarding their origin, they are most often derived from resident fibroblasts, whose transformation is induced by various cytokines or growth factors (TGF-β, FGF, PDGF, IL-1, IL-6) [112]. CAFs can also originate from the stromal cells of the bone marrow, but there are examples of their derivation from endothelial cells [113], adipose cells and pericytes [114]. In addition to the above-mentioned factors, other molecules can also promote the transformation of fibroblasts into CAFs. For instance, caveolin-deficient fibroblasts can promote the TGF–β1/Smad pathway, which aids the migration and stemness of breast cancer cells [115]. TGF-β1 is also implicated in CAF activation, which facilitates the invasion of oral cancer [116]. The term cancer-associated fibroblast does not refer to a well-defined cell type; therefore, CAFs can participate in a variety of functions, the majority of which are supportive of tumor progression. Today, at least 20 subtypes of CAFs are known, with diverse functions in various tumor types [117,118]. Despite the fact that increasing numbers of CAF subpopulations are being detected by single-cell sequencing, none of them have been assigned a strictly defined function; rather, their actions are more or less overlapping [119]. The best-known CAFs with well-defined functions are myofibroblastic CAFs (mCAFs), involved in matrix remodeling, inflammatory CAFs (iCAFs) and antigen-presenting CAFs (apCAFs) [120]. It is evident that they play a role in the overproduction of collagen, which results in stiffening of the matrix that facilitates tumor progression [121]. This situation can be exacerbated by therapeutic irradiation, which creates an additional stressor. Other factors produced by CAFs can also promote the progression of tumors [53,118]. By reorganizing the extracellular matrix and altering its structure, CAFs facilitate the migration of tumor cells. Furthermore, by causing a high connective tissue pressure, they can prevent therapeutic agents from reaching their targets. CAFs stimulate tumor angiogenesis and tumor cell proliferation by secreting growth factors (VEGF, HGF) and support tumor cell metabolism. They can regulate the innate immune response through the secretion of chemokines, cytokines and other factors, resulting in the recruitment and polarization of monocytes/macrophages and neutrophils and in a decrease in NK cell activation. CAFs can also interfere with the adaptive immune response, inhibiting the function of T cells and dendritic cells, while promoting the accumulation of immune suppressive cells such as regulatory T cells and MDSCs. They also have indirect effects on antitumor immunity via the remodeling of the ECM [53,118,122,123].

### 3.2. Cells of the Immune System

For many decades, science has faced the phenomenon that the immune defense against tumors is mostly ineffective, even when the tumor and its environment are infiltrated by a large number of inflammatory cells [124,125]. The results of research carried out over the past decades indicate that this inflammatory infiltration involves a variety of cells, including numerous cell types participating in innate and adaptive immunity, with distinct functions. While innate immunity also plays an active role in the development of a pro-tumoral state, the cellular elements of adaptive immunity, namely, B and T cells have anti-tumor potential.

#### 3.2.1. Innate Immunity

Our innate immune system is composed of a variety of cell types such as neutrophil granulocytes, monocytes–macrophages and natural killer (NK) cells, whose primary function is to defend the organism against harmful agents. However, when inflammatory processes become chronic, the body defense mechanism can go astray, and as a result of the produced cytokines, chemokines and other mediators, a mutagenic microenvironment is created, eventually promoting the development of tumors [126]. Inflammatory processes are frequently associated with tumors, and cells of the innate immune system, particularly macrophages and neutrophils, play key roles in the development and maintenance of these processes [127,128]. Besides sustaining an inflammatory environment, tumor-associated macrophages (TAMs) and neutrophils (TANs) can promote the proliferation of tumor cells, inhibit T cells and transform the microenvironment to support tumor cell invasion and angiogenesis.

Macrophages constitute a heterogeneous group of cells and are classified as M1 and M2 subtypes based on the nature of stimuli they receive, which represent the two extremes of a continuous spectrum of macrophage plasticity [129]. In the early stage of cancer development, tumors mainly contain M1-like macrophages, which are able to phagocytose tumor cells, have antigen presenting capacity and release proinflammatory factors that recruit more effector cells [130]. However, as cancer progresses, the strong tumor-supporting function of M2-like macrophages is increasingly emphasized, with a predominant role in tissue remodeling and angiogenesis, facilitating tumor progression [130,131]. Besides being able to stimulate tumor growth, angiogenesis, invasion and metastasis, TAMs also have immune inhibitory potential via the secretion of various immune-suppressive substances [130,132]. Moreover, among the tumor-associated immune cell types, macrophages highly express PD-L1, which is critical in the suppression of T cell functions [133,134]. Myeloid-derived suppressor cells (MDSCs) constitute a heterogeneous group of cells of myeloid origin, able to inhibit both the innate and the adaptive immune responses through several mechanisms such as the secretion of immune-suppressive substances (e.g., ARG1, IDO, TGF-β) and the stimulation of the expansion of regulatory T (Treg) cells [129,135]. MDSCs can differentiate into TAMs and share with TAMs many characteristics regarding immune-suppressive and other pro-tumor effects, such as the promotion of angiogenesis, EMT and cancer stemness [129].

Similarly to macrophages, neutrophil granulocytes are also capable of destroying tumor cells through the secretion of cytotoxic substances; however, they also produce growth factors, cytokines and matrix-degrading proteases, promoting tumor growth, invasion and metastasis [136,137]. In certain circumstances, neutrophils release the so-called neutrophil extracellular traps (NETs), which are web-like structures consisting of granule and cytosolic proteins assembled on decondensed chromatin [138]. Tumor-derived factors can induce NET formation, while NET deposition has several pro-tumor effects, including the stimulation of tumor cell proliferation, migration, invasion, EMT and immunosuppression, and may also contribute to the formation of the pre-metastatic niche [137,139,140].

Also belonging to the innate immune system, NK cells play an important role in the destruction of virus-infected cells and tumor cells and can also contribute to the regulation of adaptive immune reactions via interactions with antigen-presenting dendritic cells [141]. The number of tumor-infiltrating NK cells is generally low, and these cells often express low levels of activating receptors (e.g., NKp30, NKG2D) and have decreased functional activity [142].

#### 3.2.2. Adaptive (Acquired) Immunity

**Figure 2:**
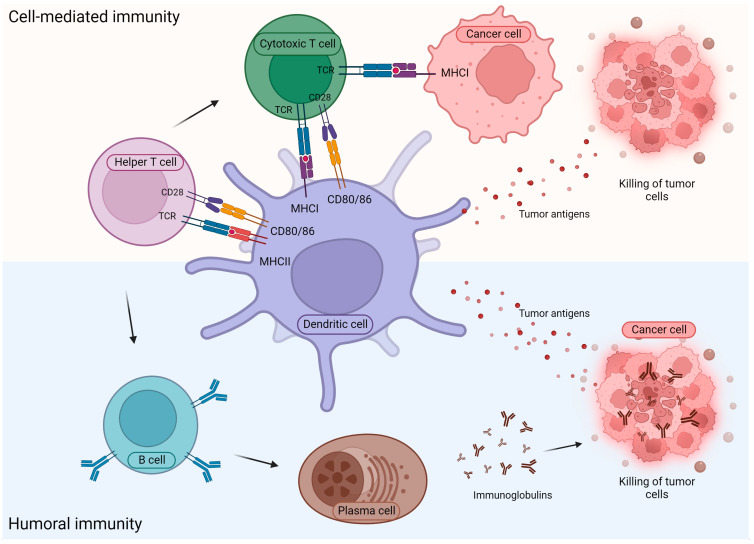
The two forms of anti-tumor acquired immune responses: cell-mediated immunity, which is based on the recognition of foreign antigens by T lymphocytes, after presentation by antigen-presenting cells, and humoral immunity, during which plasma cells produce antibodies against antigens. For explanation, see Section 3.2.2.

In contrast to innate immunity, which is not antigen-specific, adaptive immune reactions are based on the recognition of antigens by specific T or B lymphocytes. In the case of cell-mediated immunity, antigen fragments derived from foreign agents or tumor cells are presented by MHC molecules expressed in antigen-presenting cells (APCs), after processing by the antigen-processing machinery (APM), to helper (CD4+) and cytotoxic (CD8+) T lymphocytes, which recognize the cognate antigens by their specific receptors (T cell receptor, TCR) (Figure 2). Beside this stimulus, a second signal is also required for the activation of T cells, provided by costimulatory molecules. The most important costimulatory molecules are B7-1 (CD80) and B7-2 (CD86), which bind to their receptor (CD28) on the surface of T lymphocytes. Dendritic cells are known as the most efficient APCs; however, other cell types, e.g., B lymphocytes and macrophages, can also function as antigen-presenting cells.

Although solid neoplasms often contain a significant lymphoid infiltrate, tumor development and progression may occur even in the presence of antigen-specific T cells, indicating the lack of an efficient immune response. Immune-suppressive components are present in virtually all steps of the “cancer–immunity cycle” [124], for example, inhibitory immune checkpoints, cytokines and immune-suppressive enzymes, in addition to suppressor cells. Beside TAMs and MDSCs, discussed above, the best characterized suppressor cells are CD4+CD25+FOXP3+ regulatory T (Treg) cells. The physiological function of these cells is preventing autoimmune reactions, but they also inhibit antitumor immune responses through various, either contact-dependent or -independent, mechanisms, including the production of immune-suppressive cytokines and ectoenzymes, immune checkpoint interactions, etc. [143].

The other arm of adaptive immunity consists of humoral immune reactions based on the recognition of cell components by antigen-specific receptors of B lymphocytes (B cell receptors, BCRs), after they have received help from antigen-specific helper T cells. Consequently, activated B lymphocytes begin to multiply and, if necessary, differentiate into plasma cells and produce specific antibodies or transform into memory B cells [144] (Figure 2). The role of antibody-mediated immunity and B cells in general in the antitumor immune response is not clear; both anti- and pro-tumor effects have been documented [145]. There are several different subpopulations of B lymphocytes, with a diverse array of functional activities, such as antibody production, antigen presentation and the secretion of immune-suppressive cytokines. In some cases, B cells can form organized follicle-like ectopic aggregates, termed tertiary lymphoid structures (TLSs), in which the B cell zone is associated with a T cell zone and other cell types such as dendritic cells and adjacent high endothelial venules (HEVs). TLSs have been described in chronic inflammatory conditions and are also associated with tumors, where they are proposed to be sites of generation of the immune response. The presence of tumor-associated TLSs has been correlated with improved prognosis in many tumor types, but an opposite conclusion has also been reported in some cases, which could be the consequence of their different maturation state, location or cellular composition [145,146].

### 3.3. Key Players in Tumor Angiogenesis

Tumors, like healthy tissues, require blood supply. To achieve this, various tumors employ distinct strategies. They are able to create new blood vessels (endothelial sprouting), engulf existing ones (vessel co-option), partitioning existing vessels by insertion of connective tissue columns (intussusceptive microvascular growth) or stimulate the glomerular proliferation of existing vessels (glomeruloid angiogenesis) [147]. The final result is achieved under the influence of factors that stimulate or inhibit angiogenesis. The most potent and significant growth factor targeted by anti-tumor therapy is VEGF (vascular endothelial growth factor), which stimulates the proliferation of endothelial cells by binding to its receptor on the cell surface. Several additional cytokines, chemokines and growth factors are associated with the process, stimulating or inhibiting tumor vessel formation [59]. The components of tumor vessels are tumor-associated endothelial cells (TECs), pericytes and the basement membrane that supports the vessel wall. TECs are aberrant in size and shape, and their cytoplasmic projections extend across the vessel lumen and may create cracks or small intercellular spaces in the vessel wall by penetrating the lumen [148]. As a consequence, the structure of these blood vessels is defective. The blood vessel network is characterized by tortuous, dilated and frequently blind-ending arteries and capillaries, as well as by impaired microcirculation within the vasculature. The endothelial lining is also damaged. Pericytes only partially cover the outer layer of blood vessels. The basement membrane is also damaged, and its thickness and connection to the endothelial cells are irregular. As a result, tumor blood vessels are leaky. These blood vessels are not associated with lymphatic vessels. Normal fibroblasts do not produce mediators that stimulate angiogenesis, but tumor-associated fibroblasts generate bFGF, PDGF, and CXCL12 in addition to VEGF, which further support tumor neoangiogenesis. The need for nutrients of proliferating tumor cells within fast-growing malignant tumors is substantial; consequently, tumor angiogenesis has been at the forefront of research for a long time, and angiogenesis inhibitors are essential components of tumor therapies [149].

## 4. The TME as a Therapeutic Target

The accumulating knowledge about the key components of the TME and their interactions with tumor cells has enabled the development of various therapeutic strategies targeting the main cell types and molecular components of the tumor microenvironment. In the past decade, the development of a novel class of immunotherapy drugs, aiming at blocking the inhibitory mechanisms of T cell activation, opened a new front in the fight against cancer, but other cell types of the TME, as well as stromal components, are also subjects of extensive research as potential therapeutic targets.

### 4.1. Targeting the Immune System

**Figure 3:**
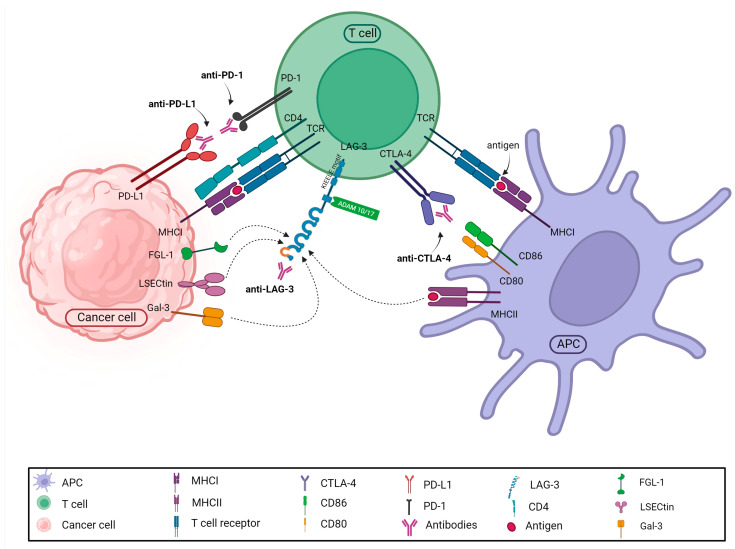
The figure presents the clinically used, approved immune checkpoint inhibitors and their target molecules. Monoclonal antibodies bind either to receptors on the T cell (e.g., anti-CTLA-4, anti-LAG-3 or anti-PD-1 antibodies) or to ligands on the surface of the tumor cells or other immune cells (anti-PD-L1 antibodies), thereby preventing ligand–receptor binding.

It has long been known that tumor cells can neutralize the effects of immune cells acting against them. However, it required many years to clarify the mechanisms of this process and develop effective medications. These so-called immune “checkpoint” inhibitors suspend immune inhibition based on the interaction between T lymphocytes expressing inhibitory immune checkpoint receptors and tumor cells or other cells expressing the corresponding ligands, thereby permitting the immune cells to destroy the tumor cells [150]. A growing number of immune checkpoint inhibitors, used in the treatment of many tumor types, are available on the market today, representing the most widely used tumor immunotherapy modality [151,152] (Figure 3).

Ipilimumab was the first, though less effective, immune checkpoint inhibitor used in the treatment of malignant melanomas. It inhibits the binding of CTLA-4 on the surface of T lymphocytes to the CD80 and CD86 molecules on APCs. A breakthrough came with the use of monoclonal antibodies against PD-1 (nivolumab, pembrolizumab, cemiplimab) or its ligand PD-L1 (atezolizumab, durvalumab, avelumab). Recently, relatlimab, an anti-LAG-3 agent, was also approved for melanoma patients in combination with nivolumab. Nowadays, research focuses on the inhibition of novel checkpoint targets, such as TIGIT, TIM-3 and VISTA [153]. Moreover, further studies are aimed at investigating agonists for costimulatory molecules, e.g., OX40 and 4-1BB [154]. The expanding understanding of acquired cellular immunity has led to the development of adoptive cell therapy (ACT) [155,156,157]. Historically, the first ACT that proved effective is tumor-infiltrating lymphocyte (TIL) therapy, reaching a high response rate in advanced melanoma patients [157]. Another approach is redirecting the specificity of T cells via genetic engineering, introducing genes encoding tumor antigen-recognizing TCRs, or transducing them with chimeric antigen receptors (CARs) [155,158]. CARs consist of an scFv fragment of a tumor antigen-specific immunoglobulin connected to the CD3ζ signaling chain via a hinge and a transmembrane domain and of costimulatory domains. CAR T cells proved highly effective mainly against hematological malignancies [159].

Immunotherapy reported many triumphs, but not all tumors respond as expected to its application [160,161]. Furthermore, the novel therapies may cause unexpected side effects such as cytokine release syndrome [162] or neurotoxicity syndrome [163]. Immunotherapy can be used as monotherapy, but there are increasing efforts to combine it with other antitumor drugs. In this regard, there have been both successes and failures, justifying the need for additional studies. An intriguing study reported that the exosomes of tumor cells were capable of inhibiting the action of natural killer cells, thereby reducing the effect of immunotherapy [164].

The efficiency of immunotherapy is influenced by tumor cell intrinsic factors, such as PD-L1 expression (in the case of PD-1/PD-L1 targeting agents), tumor mutation burden (TMB) and neoantigen and HLA expression [165,166], as well as by tumor cell extrinsic factors, derived from the TME. For immunotherapy to work, T lymphocytes must penetrate the tumor and reach the tumor cells. Accordingly, the infiltration of T lymphocytes (as well as of other immune cell types) and the expression of immune-related genes in the tumor predict response to immunotherapy [165,167]. The tumor’s immunogenicity, the local concentration of chemokines and matrix architecture affect immune cell infiltration [168,169]. The ECM influences immunotherapy efficacy in several ways [170]. A thick ECM may block the immune cells from reaching the tumor cells even in highly immunogenic malignancies. When lymphocytes meet stiff surfaces, they move along them rather than following a chemoattractive gradient, which results in an “immune-excluded” phenotype. A thickened ECM creates a diffusion barrier that may also hinder immunotherapeutic medicines such as checkpoint inhibitory antibodies from reaching the tumor. Increased hypoxia due to limited oxygen supply beyond the diffusion barrier may boost immune escape by upregulating immunomodulatory factors like IL-10 or TGF-β. In addition, hypoxia boosts angiogenic signaling. Activated blood arteries showed diminished ICAM1 expression, which prevented immune cell adhesion and extravasation [170].

Given the negative effects of the abundant and pathologically altered tumor ECM on multiple treatment modalities, there has been a clear interest in targeting the ECM to enhance the therapeutic efficacy.

### 4.2. Targeting Cancer-Associated Myeloid Cells and Fibroblasts

Altogether, tumor-associated myeloid cells comprise multiple (currently 23) subtypes, as revealed by single-cell sequencing [171]. The largest fraction is that with monocyte–macrophage origin, but dendritic and neutrophil subtypes are also present. Although anti-tumor effects have also been described, the majority of these cells have a tumor-supporting potential [172]. Based on this, many strategies have been designed to utilize them as targets for tumor therapy, attempting to prevent their recruitment or suppress their pro-tumor functions, including blocking chemokines/chemokine receptors, inducing differentiation, metabolic reprogramming or changing their phenotypic polarization [4,130,132,160,173,174,175]. Tyrosine kinase inhibitors such as sunitinib or sorafenib have also been used to deplete or repolarize myeloid cells [175]. The discovery of the anti-tumor effects of macrophages has prompted efforts to transform tumor-promoting TAMs into tumor-inhibiting M1 macrophages. The inhibition of the SIRP-1 receptor was shown to reactivate the phagocytic ability of TAMs, while the CD40 receptor-specific antibody and Toll-like receptor ligands were shown to stimulate their tumor-killing effect [176]. The inhibition of tumor intermediary metabolism can reduce the stress level in the microenvironment, thereby promoting the phenotypic transformation of TAMs [177]. Several trials are currently active aiming to inhibit tumor-associated neutrophils and NETs [140,175]. The inhibition of neutrophil extracellular traps by a paclitaxel prodrug nanoparticle core and poly-l-lysine conjugated with matrix metalloproteinase 9 (MMP-9) prevented the migration of neutrophils into the tumor microenvironment and into the tumor itself [178]. Other options, such as the reduction of hypoxia in tumors and the inhibition of transcription factors (e.g., TGF-β1), have been proposed as well. More detailed specific reviews with lists of clinical trials targeting myeloid cell types are available [175,179].

Several therapeutic attempts to inhibit CAFs have been made with varying success [4]. The discovery of novel molecules and pathways utilized by CAFs, such as integrin αvβ6 in colorectal cancer [51] or the TGF-β1/Smad2/3 signaling pathway, whose activation was shown to promote oral squamous cell carcinoma invasion [116], offers new opportunities for therapeutic interventions. Presently, many approaches aimed at specifically targeting tumor-promoting CAFs exist [119]. These tactics encompass impeding the transformation of precursor cells into tumor-promoting CAFs by the suppression of precursor activation or the targeting of crucial signaling pathways involved in the differentiation process. Another objective is to selectively eliminate CAFs while preserving tumor-restraining CAFs via genetic modification or targeted antibodies. Direct CAF depletion can be combined with CAF-induced restriction of ECM remodeling. The induction of phenotypic flipping from tumor-promoting to tumor-restraining CAFs or the disruption of the communication between cancer cells and CAFs in order to impede CAF facilitative impact on cancer cell growth, cell movement and resistance to chemotherapy are other ways to achieve effective therapies [120,123]. Indeed, a number of clinical trials targeting crucial pathways for procancerous CAF formation or maintenance, such as TGF-β, VEGF and FGF pathways, in combination with the administration of chemotherapeutic agents, demonstrated promising antitumor efficacy and tolerable safety [120]. The success of CAF-targeted preclinical studies, however, does not always guarantee beneficial effects in clinical trials, as seen in the case of pancreatic cancer patients treated with the hedgehog inhibitor IP-926 (NCT01130142) or with vismodegib (NCT01383538) in combination with gemcitabine [120].

CAFs are known to be unsusceptible to the mutagenic effects of radiotherapy, induce resistance to chemo- and targeted therapies and have a pivotal role in the development of resistance to anti-angiogenic and immune therapies. Understanding the CAF–tumor crosstalk will enable us to transform treatment strategies from a tumor-only-centered to a tumor-TME-centered approach and combine targeted therapy/immunotherapy with CAF-directed treatments to achieve favorable prognoses [119,123].

### 4.3. Targeting the Tumor Stroma

Among the components of the tumorous ECM, collagen, fibronectin, certain integrins and hyaluronic acid are expected to be of interest in terms of therapeutic intervention [4,180]. Collagen forms the structural basis of the extracellular matrix of the tumor. Accordingly, most therapeutic attempts were aimed at reducing the increased amount of collagen present in the tumor matrix, by inhibiting the factors that stimulate their production, increasing their degradation or inhibiting their cross-binding [181]. These experiments were conducted primarily in animals, but also human trials were conducted. The fungal derivative called halofungin reduced the overproduction of collagen by inhibiting TGF-β in a human breast cancer orthotopic mouse model [182], while other researchers used collagenase to reduce the stiffness of the tumor matrix for better drug penetration [183]. Looking back on the various enthusiastic attempts, it appears that a significant proportion of them have failed so far. During drug trials, novel TGF-β-inhibiting strategies demonstrated some efficacy against various solid tumors, providing an incentive for further testing [184]. Recently, inhibition of the enzyme lysyl oxidase-like 2 (LOXL2) by a humanized monoclonal antibody (simtuzumab) has been attempted in colorectal and pancreatic cancers in combination with FOLFIRI or gemcitabine, respectively [185,186]. In another study, losartan (an angiotensin II receptor antagonist) and paclitaxel packed in liposomes were administered to mice in an experimental breast cancer model, inhibiting metastasis formation. The procedure achieved a reduction in collagen and lysyl oxidase levels and also inhibited TGF-β1 [187]. It is a known fact that the CD44 HA receptor binds hyaluronic acid, which has a tumor-supporting effect [37,188]. Moreover, apoptosis is inhibited in malignancies that generate lactic acid via aerobic glycolysis. Dichloroacetate inhibits lactic acid production by reactivating the mitochondrial function, and this, in conjunction with the inhibition of hyaluronic acid synthesis by 4-methylumbelliferone, had a dual antitumor effect in the presence of aerobic glycolysis in the tumor [189].

A communication from three years ago recommending a therapeutic strategy for colorectal cancer presented an entirely novel approach [190]. In this review, the tumors were divided into four groups based on a classification of consensus molecular subtypes by an international consortium [191]. The first group (CMS1) mainly contained hypermutated MSI-positive tumors with BRAF mutation or CpG island methylation. APC-mutant hereditary tumors with activated WNT signaling and consequent Myc activation were included in the second group (CMS2). Group 3 (CMS3) tumors were distinguished by metabolic dysregulation with frequent KRAS mutations and a very poor prognosis. Group 4 (CMS4) was characterized by epithelial–mesenchymal transition, activation of TGF-β signaling, increased angiogenesis and remodeling of the matrix. The stromal classification of these subtypes also revealed differences [192]. Type 1 stroma contained many proteins that are involved in the regulation of T cells and highly differentiated Th1 and CD8+ cytotoxic cells, and the expression of CXCL13 was significant. These tumors expressed large amounts of immune checkpoint molecules (PD-1 and CTLA-4, etc.); so, immune checkpoint inhibitors were recommended for their treatment. In the stroma of group 2, few lymphocytes, macrophages, endothelial cells and fibroblasts were detected. In accordance with their known gene mutation, β-catenin inhibition was recommended. The stroma of the third group was characterized by sparse immune cell infiltration, and along with chemotherapy, an EGFR inhibitor was suggested; in the presence of KRAS mutation, angiogenesis inhibition and possibly adoptive T cell therapy were recommended. Tumors in group 4 contained immune checkpoint molecules, and their stroma was infiltrated by macrophages and myeloid-derived suppressor cells. The large amount of chemokines in their stroma induced the migration of myeloid cells. In these cases, in addition to immune checkpoint therapy, TGF-β inhibition, angiogenesis inhibition and macrophage inhibition were recommended [190]. Of course, it is again questionable how these proposals will work in the future. In any case, they reflect attempts in which the status of the stromal resident cells, cytokines, chemokines and other stromal components were considered for the selection of the therapy.

## 5. Conclusions

Although tumors originate from healthy tissues, during their malignant transformation they create their own environment, which is quantitatively and qualitatively different from the healthy tissue milieu. The tumor cell is only one component of the tumor. Next to it, virtually all types of tissue cells, such as fibroblasts, macrophages, lymphocytes and endothelial cells, line up with the initial intention of defending themselves and then adapting to changes in the circumstances. This process results in the formation of tumor-associated fibroblasts, blood vessels and immune cells with altered, tumor-supporting functions and produces an ECM that favors tumor progression. The cells that can still be activated in the body defense (e.g., CD8+ lymphocytes) are neutralized by immune checkpoint inhibitory interactions or other suppressive mechanisms. The development of immunotherapies aiming at blocking these inhibitory interactions has resulted in broadening the options of effective therapeutic modalities for many types of cancer. A precise understanding of the tumor stroma and the interactions of its cellular and molecular components is in progress and gives hope for the development of novel therapeutic strategies targeting the components of the TME and, importantly, for designing efficient, mechanism-based combination therapies.

## Data Availability

Not applicable.

## References

[1] O’Dowd E.L., Merriel S.W.D., Cheng V.W.T., Khan S., Howells L.M., Gopal D.P., Roundhill E.A., Brennan P.M., Crosbie P.A.J., Neal R.D. (2023). Clinical trials in cancer screening, prevention and early diagnosis (SPED): A systematic mapping review. BMC Cancer.

[2] Whiteside T.L. (2008). The tumor microenvironment and its role in promoting tumor growth. Oncogene.

[3] Balkwill F.R., Capasso M., Hagemann T. (2012). The tumor microenvironment at a glance. J. Cell Sci..

[4] Baghban R., Roshangar L., Jahanban-Esfahlan R., Seidi K., Ebrahimi-Kalan A., Jaymand M., Kolahian S., Javaheri T., Zare P. (2020). Tumor microenvironment complexity and therapeutic implications at a glance. Cell Commun. Signal..

[5] Rømer A.M.A., Thorseth M.L., Madsen D.H. (2021). Immune Modulatory Properties of Collagen in Cancer. Front. Immunol..

[6] Brinckerhoff C.E., Rutter J.L., Benbow U. (2000). Interstitial collagenases as markers of tumor progression. Clin. Cancer Res. Off. J. Am. Assoc. Cancer Res..

[7] Heppner K.J., Matrisian L.M., Jensen R.A., Rodgers W.H. (1996). Expression of most matrix metalloproteinase family members in breast cancer represents a tumor-induced host response. Am. J. Pathol..

[8] Chen L., Kong X., Fang Y., Paunikar S., Wang X., Brown J.A.L., Bourke E., Li X., Wang J. (2021). Recent Advances in the Role of Discoidin Domain Receptor Tyrosine Kinase 1 and Discoidin Domain Receptor Tyrosine Kinase 2 in Breast and Ovarian Cancer. Front. Cell Dev. Biol..

[9] Romayor I., García-Vaquero M.L., Márquez J., Arteta B., Barceló R., Benedicto A. (2022). Discoidin Domain Receptor 2 Expression as Worse Prognostic Marker in Invasive Breast Cancer. Breast J..

[10] Popova N.V., Jücker M. (2022). The Functional Role of Extracellular Matrix Proteins in Cancer. Cancers.

[11] Lin T.-C., Yang C.-H., Cheng L.-H., Chang W.-T., Lin Y.-R., Cheng H.-C. (2020). Fibronectin in Cancer: Friend or Foe. Cells.

[12] Erdogan B., Ao M., White L.M., Means A.L., Brewer B.M., Yang L., Washington M.K., Shi C., Franco O.E., Weaver A.M. (2017). Cancer-associated fibroblasts promote directional cancer cell migration by aligning fibronectin. J. Cell Biol..

[13] Gordon-Weeks A., Lim S.Y., Yuzhalin A., Lucotti S., Vermeer J.A.F., Jones K., Chen J., Muschel R.J. (2019). Tumour-Derived Laminin α5 (LAMA5) Promotes Colorectal Liver Metastasis Growth, Branching Angiogenesis and Notch Pathway Inhibition. Cancers.

[14] Hohenester E., Yurchenco P.D. (2013). Laminins in basement membrane assembly. Cell Adhes. Migr..

[15] Giannelli G., Antonaci S. (2000). Biological and clinical relevance of Laminin-5 in cancer. Clin. Exp. Metastasis.

[16] Miyazaki K. (2006). Laminin-5 (laminin-332): Unique biological activity and role in tumor growth and invasion. Cancer Sci..

[17] Fullár A., Dudás J., Oláh L., Hollósi P., Papp Z., Sobel G., Karászi K., Paku S., Baghy K., Kovalszky I. (2015). Remodeling of extracellular matrix by normal and tumor-associated fibroblasts promotes cervical cancer progression. BMC Cancer.

[18] Daisuke H., Kato H., Fukumura K., Mayeda A., Miyagi Y., Seiki M., Koshikawa N. (2021). Novel LAMC2 fusion protein has tumor-promoting properties in ovarian carcinoma. Cancer Sci..

[19] Iozzo R.V., Schaefer L. (2015). Proteoglycan form and function: A comprehensive nomenclature of proteoglycans. Matrix Biol. J. Int. Soc. Matrix Biol..

[20] Karamanos N.K., Theocharis A.D., Neill T., Iozzo R.V. (2019). Matrix modeling and remodeling: A biological interplay regulating tissue homeostasis and diseases. Matrix Biol. J. Int. Soc. Matrix Biol..

[21] Karamanos N.K., Theocharis A.D., Piperigkou Z., Manou D., Passi A., Skandalis S.S., Vynios D.H., Orian-Rousseau V., Ricard-Blum S., Schmelzer C.E.H. (2021). A guide to the composition and functions of the extracellular matrix. FEBS J..

[22] Theocharis A.D., Manou D., Karamanos N.K. (2019). The extracellular matrix as a multitasking player in disease. FEBS J..

[23] Reszegi A., Horváth Z., Karászi K., Regős E., Postniková V., Tátrai P., Kiss A., Schaff Z., Kovalszky I., Baghy K. (2020). The Protective Role of Decorin in Hepatic Metastasis of Colorectal Carcinoma. Biomolecules.

[24] Zheng X., Liu X., Lei Y., Wang G., Liu M. (2022). Glypican-3: A Novel and Promising Target for the Treatment of Hepatocellular Carcinoma. Front. Oncol..

[25] Chakraborty S., Hong W. (2018). Linking Extracellular Matrix Agrin to the Hippo Pathway in Liver Cancer and Beyond. Cancers.

[26] Váncza L., Karászi K., Péterfia B., Turiák L., Dezső K., Sebestyén A., Reszegi A., Petővári G., Kiss A., Schaff Z. (2022). SPOCK1 Promotes the Development of Hepatocellular Carcinoma. Front. Oncol..

[27] Stanford K.I., Bishop J.R., Foley E.M., Gonzales J.C., Niesman I.R., Witztum J.L., Esko J.D. (2009). Syndecan-1 is the primary heparan sulfate proteoglycan mediating hepatic clearance of triglyceride-rich lipoproteins in mice. J. Clin. Investig..

[28] Regős E., Karászi K., Reszegi A., Kiss A., Schaff Z., Baghy K., Kovalszky I. (2020). Syndecan-1 in Liver Diseases. Pathol. Oncol. Res..

[29] Inki P., Larjava H., Haapasalmi K., Miettinen H.M., Grenman R., Jalkanen M. (1994). Expression of syndecan-1 is induced by differentiation and suppressed by malignant transformation of human keratinocytes. Eur. J. Cell Biol..

[30] Inki P., Jalkanen M. (1996). The role of syndecan-1 in malignancies. Ann. Med..

[31] Sanderson R.D., Børset M. (2002). Syndecan-1 in B lymphoid malignancies. Ann. Hematol..

[32] Kind S., Jaretzke A., Büscheck F., Möller K., Dum D., Höflmayer D., Hinsch A., Weidemann S., Fraune C., Möller-Koop C. (2019). A shift from membranous and stromal syndecan-1 (CD138) expression to cytoplasmic CD138 expression is associated with poor prognosis in breast cancer. Mol. Carcinog..

[33] Kind S., Kluth M., Hube-Magg C., Möller K., Makrypidi-Fraune G., Lutz F., Lennartz M., Rico S.D., Schlomm T., Heinzer H. (2020). Increased Cytoplasmic CD138 Expression Is Associated with Aggressive Characteristics in Prostate Cancer and Is an Independent Predictor for Biochemical Recurrence. BioMed Res. Int..

[34] Couchman J.R. (2021). Syndecan-1 (CD138), Carcinomas and EMT. Int. J. Mol. Sci..

[35] Kind S., Merenkow C., Büscheck F., Möller K., Dum D., Chirico V., Luebke A.M., Höflmayer D., Hinsch A., Jacobsen F. (2019). Prevalence of Syndecan-1 (CD138) Expression in Different Kinds of Human Tumors and Normal Tissues. Dis. Markers.

[36] Iaconisi G.N., Lunetti P., Gallo N., Cappello A.R., Fiermonte G., Dolce V., Capobianco L. (2023). Hyaluronic Acid: A Powerful Biomolecule with Wide-Ranging Applications—A Comprehensive Review. Int. J. Mol. Sci..

[37] Tavianatou A.G., Caon I., Franchi M., Piperigkou Z., Galesso D., Karamanos N.K. (2019). Hyaluronan: Molecular size-dependent signaling and biological functions in inflammation and cancer. FEBS J..

[38] Barczyk M., Carracedo S., Gullberg D. (2010). Integrins. Cell Tissue Res..

[39] Eble J.A., Gullberg D. (2019). What Is the Fuss about Integrins and the Tumor Microenvironment?. Cancers.

[40] Desgrosellier J.S., Cheresh D.A. (2010). Integrins in cancer: Biological implications and therapeutic opportunities. Nat. Rev. Cancer.

[41] Pytela R., Pierschbacher M.D., Ruoslahti E. (1985). Identification and isolation of a 140 kd cell surface glycoprotein with properties expected of a fibronectin receptor. Cell.

[42] Berrier A.L., Yamada K.M. (2007). Cell-matrix adhesion. J. Cell. Physiol..

[43] Munksgaard Thorén M., Chmielarska Masoumi K., Krona C., Huang X., Kundu S., Schmidt L., Forsberg-Nilsson K., Floyd Keep M., Englund E., Nelander S. (2019). Integrin α10, a Novel Therapeutic Target in Glioblastoma, Regulates Cell Migration, Proliferation, and Survival. Cancers.

[44] Zeltz C., Pasko E., Cox T.R., Navab R., Tsao M.S. (2019). LOXL1 Is Regulated by Integrin α11 and Promotes Non-Small Cell Lung Cancer Tumorigenicity. Cancers.

[45] Martínez-Nieto G.A., Teppo H.R., Petrelius N., Izzi V., Devarajan R., Petäistö T., Liu H., Kim K.S., Karppinen S.M., Ruotsalainen H. (2022). Upregulated integrin α11 in the stroma of cutaneous squamous cell carcinoma promotes skin carcinogenesis. Front. Oncol..

[46] Zeltz C., Lu N., Heljasvaara R., Gullberg D., Kovalszky I., Franchi M., Alaniz L.D. (2022). Integrins in Cancer: Refocusing on the Tumor Microenvironment. The Extracellular Matrix and the Tumor Microenvironment.

[47] Zeltz C., Alam J., Liu H., Erusappan P.M., Hoschuetzky H., Molven A., Parajuli H., Cukierman E., Costea D.E., Lu N. (2019). α11β1 Integrin is Induced in a Subset of Cancer-Associated Fibroblasts in Desmoplastic Tumor Stroma and Mediates In Vitro Cell Migration. Cancers.

[48] Cavaco A.C.M., Rezaei M., Caliandro M.F., Lima A.M., Stehling M., Dhayat S.A., Haier J., Brakebusch C., Eble J.A. (2018). The Interaction between Laminin-332 and α3β1 Integrin Determines Differentiation and Maintenance of CAFs, and Supports Invasion of Pancreatic Duct Adenocarcinoma Cells. Cancers.

[49] Böger C., Warneke V.S., Behrens H.M., Kalthoff H., Goodman S.L., Becker T., Röcken C. (2015). Integrins αvβ3 and αvβ5 as prognostic, diagnostic, and therapeutic targets in gastric cancer. Gastric Cancer Off. J. Int. Gastric Cancer Assoc. Jpn. Gastric Cancer Assoc..

[50] Breuss J.M., Gallo J., DeLisser H.M., Klimanskaya I.V., Folkesson H.G., Pittet J.F., Nishimura S.L., Aldape K., Landers D.V., Carpenter W. (1995). Expression of the beta 6 integrin subunit in development, neoplasia and tissue repair suggests a role in epithelial remodeling. J. Cell Sci..

[51] Peng C., Zou X., Xia W., Gao H., Li Z., Liu N., Xu Z., Gao C., He Z., Niu W. (2018). Integrin αvβ6 plays a bi-directional regulation role between colon cancer cells and cancer-associated fibroblasts. Biosci. Rep..

[52] Zhao H.Q., Jiang J. (2023). Chemokines and receptors in the development and progression of malignant tumors. Cytokine.

[53] Shah K., Mallik S.B., Gupta P., Iyer A. (2022). Targeting Tumour-Associated Fibroblasts in Cancers. Front. Oncol..

[54] Tang P.W., Frisbie L., Hempel N., Coffman L. (2023). Insights into the tumor-stromal-immune cell metabolism cross talk in ovarian cancer. Am. J. Physiol. Cell Physiol..

[55] Choi H.Y., Chang J.E. (2023). Targeted Therapy for Cancers: From Ongoing Clinical Trials to FDA-Approved Drugs. Int. J. Mol. Sci..

[56] Wang Q., Xiong F., Wu G., Wang D., Liu W., Chen J., Qi Y., Wang B., Chen Y. (2023). SMAD Proteins in TGF-β Signalling Pathway in Cancer: Regulatory Mechanisms and Clinical Applications. Diagnostics.

[57] Jariwala N., Ozols M., Bell M., Bradley E., Gilmore A., Debelle L., Sherratt M.J. (2022). Matrikines as mediators of tissue remodelling. Adv. Drug Deliv. Rev..

[58] Mongiat M., Sweeney S.M., San Antonio J.D., Fu J., Iozzo R.V. (2003). Endorepellin, a novel inhibitor of angiogenesis derived from the C terminus of perlecan. J. Biol. Chem..

[59] Kovalszky I., Váncza L., Reszegi A., Tátrai P., Baghy K., Kovalszky I., Franchi M., Alaniz L.D. (2022). Cancer Angiogenesis and Its Master Regulator Perlecan. The Extracellular Matrix and the Tumor Microenvironment.

[60] Papadas A., Arauz G., Cicala A., Wiesner J., Asimakopoulos F. (2020). Versican and Versican-matrikines in Cancer Progression, Inflammation, and Immunity. J. Histochem. Cytochem. Off. J. Histochem. Soc..

[61] Vizovisek M., Ristanovic D., Menghini S., Christiansen M.G., Schuerle S. (2021). The Tumor Proteolytic Landscape: A Challenging Frontier in Cancer Diagnosis and Therapy. Int. J. Mol. Sci..

[62] Duffy M.J., Maguire T.M., Hill A., McDermott E., O’Higgins N. (2000). Metalloproteinases: Role in breast carcinogenesis, invasion and metastasis. Breast Cancer Res..

[63] Mitschke J., Burk U.C., Reinheckel T. (2019). The role of proteases in epithelial-to-mesenchymal cell transitions in cancer. Cancer Metastasis Rev..

[64] Atkinson J.M., Siller C.S., Gill J.H. (2008). Tumour endoproteases: The cutting edge of cancer drug delivery?. Br. J. Pharmacol..

[65] Rappay G. (1989). Proteinases and their inhibitors in cells and tissues. Prog. Histochem. Cytochem..

[66] Westermarck J., Kähäri V.M. (1999). Regulation of matrix metalloproteinase expression in tumor invasion. FASEB J. Off. Publ. Fed. Am. Soc. Exp. Biol..

[67] Alaseem A., Alhazzani K., Dondapati P., Alobid S., Bishayee A., Rathinavelu A. (2019). Matrix Metalloproteinases: A challenging paradigm of cancer management. Semin. Cancer Biol..

[68] Klein T., Bischoff R. (2011). Physiology and pathophysiology of matrix metalloproteases. Amino Acids.

[69] Jabłońska-Trypuć A., Matejczyk M., Rosochacki S. (2016). Matrix metalloproteinases (MMPs), the main extracellular matrix (ECM) enzymes in collagen degradation, as a target for anticancer drugs. J. Enzym. Inhib. Med. Chem..

[70] Jackson H.W., Defamie V., Waterhouse P., Khokha R. (2017). TIMPs: Versatile extracellular regulators in cancer. Nat. Rev. Cancer.

[71] Brew K., Nagase H. (2010). The tissue inhibitors of metalloproteinases (TIMPs): An ancient family with structural and functional diversity. Biochim. Biophys. Acta.

[72] Fanjul-Fernández M., Folgueras A.R., Cabrera S., López-Otín C. (2010). Matrix metalloproteinases: Evolution, gene regulation and functional analysis in mouse models. Biochim. Biophys. Acta.

[73] Egeblad M., Werb Z. (2002). New functions for the matrix metalloproteinases in cancer progression. Nat. Rev. Cancer.

[74] Solanas G., Cortina C., Sevillano M., Batlle E. (2011). Cleavage of E-cadherin by ADAM10 mediates epithelial cell sorting downstream of EphB signalling. Nat. Cell Biol..

[75] Murai T., Miyauchi T., Yanagida T., Sako Y. (2006). Epidermal growth factor-regulated activation of Rac GTPase enhances CD44 cleavage by metalloproteinase disintegrin ADAM10. Biochem. J..

[76] Mullooly M., McGowan P.M., Crown J., Duffy M.J. (2016). The ADAMs family of proteases as targets for the treatment of cancer. Cancer Biol. Ther..

[77] Weber S., Saftig P. (2012). Ectodomain shedding and ADAMs in development. Development.

[78] Huovila A.P., Turner A.J., Pelto-Huikko M., Kärkkäinen I., Ortiz R.M. (2005). Shedding light on ADAM metalloproteinases. Trends Biochem. Sci..

[79] Sahin U., Weskamp G., Kelly K., Zhou H.M., Higashiyama S., Peschon J., Hartmann D., Saftig P., Blobel C.P. (2004). Distinct roles for ADAM10 and ADAM17 in ectodomain shedding of six EGFR ligands. J. Cell Biol..

[80] Cal S., López-Otín C. (2015). ADAMTS proteases and cancer. Matrix Biol..

[81] Wagstaff L., Kelwick R., Decock J., Edwards D.R. (2011). The roles of ADAMTS metalloproteinases in tumorigenesis and metastasis. Front. Biosci. (Landmark Ed.).

[82] Stanton H., Melrose J., Little C.B., Fosang A.J. (2011). Proteoglycan degradation by the ADAMTS family of proteinases. Biochim. Biophys. Acta.

[83] Kelwick R., Desanlis I., Wheeler G.N., Edwards D.R. (2015). The ADAMTS (A Disintegrin and Metalloproteinase with Thrombospondin motifs) family. Genome Biol..

[84] Unruh D., Horbinski C. (2020). Beyond thrombosis: The impact of tissue factor signaling in cancer. J. Hematol. Oncol..

[85] Chowdhury D., Lieberman J. (2008). Death by a thousand cuts: Granzyme pathways of programmed cell death. Annu. Rev. Immunol..

[86] Kristensen J.H., Karsdal M.A., Sand J.M., Willumsen N., Diefenbach C., Svensson B., Hägglund P., Oersnes-Leeming D.J. (2015). Serological assessment of neutrophil elastase activity on elastin during lung ECM remodeling. BMC Pulm. Med..

[87] Wada Y., Yoshida K., Tsutani Y., Shigematsu H., Oeda M., Sanada Y., Suzuki T., Mizuiri H., Hamai Y., Tanabe K. (2007). Neutrophil elastase induces cell proliferation and migration by the release of TGF-alpha, PDGF and VEGF in esophageal cell lines. Oncol. Rep..

[88] Julier Z., Martino M.M., de Titta A., Jeanbart L., Hubbell J.A. (2015). The TLR4 agonist fibronectin extra domain A is cryptic, exposed by elastase-2; use in a fibrin matrix cancer vaccine. Sci. Rep..

[89] Wilson T.J., Nannuru K.C., Singh R.K. (2009). Cathepsin G-mediated activation of pro-matrix metalloproteinase 9 at the tumor-bone interface promotes transforming growth factor-beta signaling and bone destruction. Mol. Cancer Res..

[90] Wilson T.J., Nannuru K.C., Futakuchi M., Singh R.K. (2010). Cathepsin G-mediated enhanced TGF-beta signaling promotes angiogenesis via upregulation of VEGF and MCP-1. Cancer Lett..

[91] Yui S., Osawa Y., Ichisugi T., Morimoto-Kamata R. (2014). Neutrophil cathepsin G, but not elastase, induces aggregation of MCF-7 mammary carcinoma cells by a protease activity-dependent cell-oriented mechanism. Mediat. Inflamm..

[92] Morimoto-Kamata R., Yui S. (2017). Insulin-like growth factor-1 signaling is responsible for cathepsin G-induced aggregation of breast cancer MCF-7 cells. Cancer Sci..

[93] Kramer L., Turk D., Turk B. (2017). The Future of Cysteine Cathepsins in Disease Management. Trends Pharmacol. Sci..

[94] Olson O.C., Joyce J.A. (2015). Cysteine cathepsin proteases: Regulators of cancer progression and therapeutic response. Nat. Rev. Cancer.

[95] Ramirez M.L.G., Salvesen G.S. (2018). A primer on caspase mechanisms. Semin. Cell Dev. Biol..

[96] Mohammad R.M., Muqbil I., Lowe L., Yedjou C., Hsu H.Y., Lin L.T., Siegelin M.D., Fimognari C., Kumar N.B., Dou Q.P. (2015). Broad targeting of resistance to apoptosis in cancer. Semin. Cancer Biol..

[97] Boice A., Bouchier-Hayes L. (2020). Targeting apoptotic caspases in cancer. Biochim. Biophys. Acta. Mol. Cell Res..

[98] Storr S.J., Carragher N.O., Frame M.C., Parr T., Martin S.G. (2011). The calpain system and cancer. Nat. Rev. Cancer.

[99] Chen B., Tang J., Guo Y.S., Li Y., Chen Z.N., Jiang J.L. (2013). Calpains are required for invasive and metastatic potentials of human HCC cells. Cell Biol. Int..

[100] Wegman-Ostrosky T., Soto-Reyes E., Vidal-Millán S., Sánchez-Corona J. (2015). The renin-angiotensin system meets the hallmarks of cancer. J. Renin-Angiotensin-Aldosterone Syst..

[101] George A.J., Thomas W.G., Hannan R.D. (2010). The renin-angiotensin system and cancer: Old dog, new tricks. Nat. Rev. Cancer.

[102] Nakamura K., Yaguchi T., Ohmura G., Kobayashi A., Kawamura N., Iwata T., Kiniwa Y., Okuyama R., Kawakami Y. (2018). Involvement of local renin-angiotensin system in immunosuppression of tumor microenvironment. Cancer Sci..

[103] Berchem G., Glondu M., Gleizes M., Brouillet J.P., Vignon F., Garcia M., Liaudet-Coopman E. (2002). Cathepsin-D affects multiple tumor progression steps in vivo: Proliferation, angiogenesis and apoptosis. Oncogene.

[104] Zaidi N., Hermann C., Herrmann T., Kalbacher H. (2008). Emerging functional roles of cathepsin E. Biochem. Biophys. Res. Commun..

[105] Zaidi N., Kalbacher H. (2008). Cathepsin E: A mini review. Biochem. Biophys. Res. Commun..

[106] Shen S., Jiang J., Yuan Y. (2017). Pepsinogen C expression, regulation and its relationship with cancer. Cancer Cell Int..

[107] Brasch F., Ochs M., Kahne T., Guttentag S., Schauer-Vukasinovic V., Derrick M., Johnen G., Kapp N., Muller K.M., Richter J. (2003). Involvement of napsin A in the C- and N-terminal processing of surfactant protein B in type-II pneumocytes of the human lung. J. Biol. Chem..

[108] Stoll L.M., Johnson M.W., Gabrielson E., Askin F., Clark D.P., Li Q.K. (2010). The utility of napsin-A in the identification of primary and metastatic lung adenocarcinoma among cytologically poorly differentiated carcinomas. Cancer Cytopathol..

[109] Collins G.A., Goldberg A.L. (2017). The Logic of the 26S Proteasome. Cell.

[110] Manasanch E.E., Orlowski R.Z. (2017). Proteasome inhibitors in cancer therapy. Nat. Rev. Clin. Oncol..

[111] Chen D., Frezza M., Schmitt S., Kanwar J., Dou Q.P. (2011). Bortezomib as the first proteasome inhibitor anticancer drug: Current status and future perspectives. Curr. Cancer Drug Targets.

[112] Park D., Sahai E., Rullan A. (2020). SnapShot: Cancer-Associated Fibroblasts. Cell.

[113] Sobierajska K., Ciszewski W.M., Sacewicz-Hofman I., Niewiarowska J. (2020). Endothelial Cells in the Tumor Microenvironment. Adv. Exp. Med. Biol..

[114] Liu T., Han C., Wang S., Fang P., Ma Z., Xu L., Yin R. (2019). Cancer-associated fibroblasts: An emerging target of anti-cancer immunotherapy. J. Hematol. Oncol..

[115] Huang Q., Wu L., Wang Y., Kong X., Xiao X., Huang Q., Li M., Zhai Y., Shi F., Zhao R. (2022). Caveolin-1-deficient fibroblasts promote migration, invasion, and stemness via activating the TGF-β/Smad signaling pathway in breast cancer cells. Acta Biochim. Biophys. Sin..

[116] Yang W., Zhang S., Li T., Zhou Z., Pan J. (2022). Single-cell analysis reveals that cancer-associated fibroblasts stimulate oral squamous cell carcinoma invasion via the TGF-β/Smad pathway. Acta Biochim. Biophys. Sin..

[117] Costa A., Kieffer Y., Scholer-Dahirel A., Pelon F., Bourachot B., Cardon M., Sirven P., Magagna I., Fuhrmann L., Bernard C. (2018). Fibroblast Heterogeneity and Immunosuppressive Environment in Human Breast Cancer. Cancer Cell.

[118] Chen Y., McAndrews K.M., Kalluri R. (2021). Clinical and therapeutic relevance of cancer-associated fibroblasts. Nat. Rev. Clin. Oncol..

[119] De P., Aske J., Dey N. (2021). Cancer-Associated Fibroblast Functions as a Road-Block in Cancer Therapy. Cancers.

[120] Yang D., Liu J., Qian H., Zhuang Q. (2023). Cancer-associated fibroblasts: From basic science to anticancer therapy. Exp. Mol. Med..

[121] Calvo F., Ege N., Grande-Garcia A., Hooper S., Jenkins R.P., Chaudhry S.I., Harrington K., Williamson P., Moeendarbary E., Charras G. (2013). Mechanotransduction and YAP-dependent matrix remodelling is required for the generation and maintenance of cancer-associated fibroblasts. Nat. Cell Biol..

[122] Ziani L., Chouaib S., Thiery J. (2018). Alteration of the Antitumor Immune Response by Cancer-Associated Fibroblasts. Front. Immunol..

[123] Zhang C., Fei Y., Wang H., Hu S., Liu C., Hu R., Du Q. (2023). CAFs orchestrates tumor immune microenvironment—A new target in cancer therapy?. Front. Pharmacol..

[124] Chen D.S., Mellman I. (2013). Oncology Meets Immunology: The Cancer-Immunity Cycle. Immunity.

[125] Stewart T.J., Smyth M.J. (2011). Improving cancer immunotherapy by targeting tumor-induced immune suppression. Cancer Metastasis Rev..

[126] Khramtsov V.V., Gillies R.J. (2014). Janus-faced tumor microenvironment and redox. Antioxid. Redox Signal..

[127] Coussens L.M., Werb Z. (2002). Inflammation and cancer. Nature.

[128] Solinas G., Marchesi F., Garlanda C., Mantovani A., Allavena P. (2010). Inflammation-mediated promotion of invasion and metastasis. Cancer Metastasis Rev..

[129] Ugel S., De Sanctis F., Mandruzzato S., Bronte V. (2015). Tumor-induced myeloid deviation: When myeloid-derived suppressor cells meet tumor-associated macrophages. J. Clin. Investig..

[130] Degboé Y., Poupot R., Poupot M. (2022). Repolarization of Unbalanced Macrophages: Unmet Medical Need in Chronic Inflammation and Cancer. Int. J. Mol. Sci..

[131] Wang Y., Lin Y.X., Qiao S.L., Wang J., Wang H. (2019). Progress in Tumor-Associated Macrophages: From Bench to Bedside. Adv. Biosyst..

[132] Mantovani A., Marchesi F., Jaillon S., Garlanda C., Allavena P. (2021). Tumor-associated myeloid cells: Diversity and therapeutic targeting. Cell. Mol. Immunol..

[133] Liu Y., Zugazagoitia J., Ahmed F.S., Henick B.S., Gettinger S.N., Herbst R.S., Schalper K.A., Rimm D.L. (2020). Immune Cell PD-L1 Colocalizes with Macrophages and Is Associated with Outcome in PD-1 Pathway Blockade Therapy. Clin. Cancer Res. Off. J. Am. Assoc. Cancer Res..

[134] Petty A.J., Dai R., Lapalombella R., Baiocchi R.A., Benson D.M., Li Z., Huang X., Yang Y. (2021). Hedgehog-induced PD-L1 on tumor-associated macrophages is critical for suppression of tumor-infiltrating CD8+ T cell function. JCI Insight.

[135] Gabrilovich D.I. (2017). Myeloid-Derived Suppressor Cells. Cancer Immunol. Res..

[136] Piccard H., Muschel R.J., Opdenakker G. (2012). On the dual roles and polarized phenotypes of neutrophils in tumor development and progression. Crit. Rev. Oncol./Hematol..

[137] Kaltenmeier C., Simmons R.L., Tohme S., Yazdani H.O. (2021). Neutrophil Extracellular Traps (NETs) in Cancer Metastasis. Cancers.

[138] Papayannopoulos V. (2018). Neutrophil extracellular traps in immunity and disease. Nat. Rev. Immunol..

[139] De Meo M.L., Spicer J.D. (2021). The role of neutrophil extracellular traps in cancer progression and metastasis. Semin. Immunol..

[140] Chen Y., Hu H., Tan S., Dong Q., Fan X., Wang Y., Zhang H., He J. (2022). The role of neutrophil extracellular traps in cancer progression, metastasis and therapy. Exp. Hematol. Oncol..

[141] Degli-Esposti M.A., Smyth M.J. (2005). Close encounters of different kinds: Dendritic cells and NK cells take centre stage. Nat. Rev. Immunol..

[142] Platonova S., Cherfils-Vicini J., Damotte D., Crozet L., Vieillard V., Validire P., André P., Dieu-Nosjean M.C., Alifano M., Régnard J.F. (2011). Profound coordinated alterations of intratumoral NK cell phenotype and function in lung carcinoma. Cancer Res..

[143] Grover P., Goel P.N., Greene M.I. (2021). Regulatory T Cells: Regulation of Identity and Function. Front. Immunol..

[144] Akkaya M., Kwak K., Pierce S.K. (2020). B cell memory: Building two walls of protection against pathogens. Nat. Rev. Immunol..

[145] Fridman W.H., Meylan M., Petitprez F., Sun C.M., Italiano A., Sautès-Fridman C. (2022). B cells and tertiary lymphoid structures as determinants of tumour immune contexture and clinical outcome. Nat. Rev. Clin. Oncol..

[146] Colbeck E.J., Ager A., Gallimore A., Jones G.W. (2017). Tertiary Lymphoid Structures in Cancer: Drivers of Antitumor Immunity, Immunosuppression, or Bystander Sentinels in Disease?. Front. Immunol..

[147] Döme B., Hendrix M.J., Paku S., Tóvári J., Tímár J. (2007). Alternative vascularization mechanisms in cancer: Pathology and therapeutic implications. Am. J. Pathol..

[148] Dudley A.C. (2012). Tumor endothelial cells. Cold Spring Harb. Perspect. Med..

[149] Lugano R., Ramachandran M., Dimberg A. (2020). Tumor angiogenesis: Causes, consequences, challenges and opportunities. Cell. Mol. Life Sci..

[150] Pardoll D.M. (2012). The blockade of immune checkpoints in cancer immunotherapy. Nat. Rev. Cancer.

[151] Sharma P., Siddiqui B.A., Anandhan S., Yadav S.S., Subudhi S.K., Gao J., Goswami S., Allison J.P. (2021). The Next Decade of Immune Checkpoint Therapy. Cancer Discov..

[152] Marei H.E., Hasan A., Pozzoli G., Cenciarelli C. (2023). Cancer immunotherapy with immune checkpoint inhibitors (ICIs): Potential, mechanisms of resistance, and strategies for reinvigorating T cell responsiveness when resistance is acquired. Cancer Cell Int..

[153] Ziogas D.C., Theocharopoulos C., Lialios P.P., Foteinou D., Koumprentziotis I.A., Xynos G., Gogas H. (2023). Beyond CTLA-4 and PD-1 Inhibition: Novel Immune Checkpoint Molecules for Melanoma Treatment. Cancers.

[154] Müller D. (2023). Targeting Co-Stimulatory Receptors of the TNF Superfamily for Cancer Immunotherapy. BioDrugs Clin. Immunother. Biopharm. Gene Ther..

[155] Lei K., Kurum A., Tang L. (2020). Mechanical Immunoengineering of T cells for Therapeutic Applications. Acc. Chem. Res..

[156] Mochel J.P., Ekker S.C., Johannes C.M., Jergens A.E., Allenspach K., Bourgois-Mochel A., Knouse M., Benzekry S., Wierson W., LeBlanc A.K. (2019). CAR T Cell Immunotherapy in Human and Veterinary Oncology: Changing the Odds Against Hematological Malignancies. AAPS J..

[157] Rosenberg S.A., Restifo N.P. (2015). Adoptive cell transfer as personalized immunotherapy for human cancer. Science.

[158] Quinn S., Lenart N., Dronzek V., Scurti G.M., Hossain N.M., Nishimura M.I. (2022). Genetic Modification of T Cells for the Immunotherapy of Cancer. Vaccines.

[159] Haslauer T., Greil R., Zaborsky N., Geisberger R. (2021). CAR T-Cell Therapy in Hematological Malignancies. Int. J. Mol. Sci..

[160] Madden D.L. (2018). From a Patient Advocate’s Perspective: Does Cancer Immunotherapy Represent a Paradigm Shift?. Curr. Oncol. Rep..

[161] Munro N. (2019). Immunology and Immunotherapy in Critical Care: An Overview. AACN Adv. Crit. Care.

[162] Smith L., Venella K. (2017). Cytokine Release Syndrome: Inpatient Care for Side Effects of CAR T-Cell Therapy. Clin. J. Oncol. Nurs..

[163] Huang S., de Jong D., Das J.P., Widemon R.S., Braumuller B., Paily J., Deng A., Liou C., Roa T., Huang A. (2023). Imaging the Side Effects of CAR T Cell Therapy: A Primer for the Practicing Radiologist. Acad. Radiol..

[164] Yu H., Huang T., Wang D., Chen L., Lan X., Liu X., Chen K., He H., Li S., Zhou Y. (2021). Acute lymphoblastic leukemia-derived exosome inhibits cytotoxicity of natural killer cells by TGF-β signaling pathway. 3 Biotech.

[165] Sharma P., Hu-Lieskovan S., Wargo J.A., Ribas A. (2017). Primary, Adaptive, and Acquired Resistance to Cancer Immunotherapy. Cell.

[166] Ladányi A., Papp E., Mohos A., Balatoni T., Liszkay G., Oláh J., Varga A., Lengyel Z., Emri G., Ferrone S. (2020). Role of the anatomic site in the association of HLA class I antigen expression level in metastases with clinical response to ipilimumab therapy in patients with melanoma. J. Immunother. Cancer.

[167] Balatoni T., Mohos A., Papp E., Sebestyén T., Liszkay G., Oláh J., Varga A., Lengyel Z., Emri G., Gaudi I. (2018). Tumor-infiltrating immune cells as potential biomarkers predicting response to treatment and survival in patients with metastatic melanoma receiving ipilimumab therapy. Cancer Immunol. Immunother. CII.

[168] Hallmann R., Zhang X., Di Russo J., Li L., Song J., Hannocks M.J., Sorokin L. (2015). The regulation of immune cell trafficking by the extracellular matrix. Curr. Opin. Cell Biol..

[169] Salmon H., Franciszkiewicz K., Damotte D., Dieu-Nosjean M.C., Validire P., Trautmann A., Mami-Chouaib F., Donnadieu E. (2012). Matrix architecture defines the preferential localization and migration of T cells into the stroma of human lung tumors. J. Clin. Investig..

[170] Henke E., Nandigama R., Ergün S. (2019). Extracellular Matrix in the Tumor Microenvironment and Its Impact on Cancer Therapy. Front. Mol. Biosci..

[171] Wang X., Xu Y., Sun Q., Zhou X., Ma W., Wu J., Zhuang J., Sun C. (2022). New insights from the single-cell level: Tumor associated macrophages heterogeneity and personalized therapy. Biomed. Pharmacother..

[172] Weissleder R., Pittet M.J. (2020). The expanding landscape of inflammatory cells affecting cancer therapy. Nat. Biomed. Eng..

[173] Cao Y., Qiao B., Chen Q., Xie Z., Dou X., Xu L., Ran H., Zhang L., Wang Z. (2023). Tumor microenvironment remodeling via targeted depletion of M2-like tumor-associated macrophages for cancer immunotherapy. Acta Biomater..

[174] Wang Y., Barrett A., Hu Q. (2023). Targeting Macrophages for Tumor Therapy. AAPS J..

[175] Cao J., Chow L., Dow S. (2023). Strategies to overcome myeloid cell induced immune suppression in the tumor microenvironment. Front. Oncol..

[176] Andrejeva G., Capoccia B.J., Hiebsch R.R., Donio M.J., Darwech I.M., Puro R.J., Pereira D.S. (2021). Novel SIRPα Antibodies That Induce Single-Agent Phagocytosis of Tumor Cells while Preserving T Cells. J. Immunol..

[177] Mojsilovic S.S., Mojsilovic S., Villar V.H., Santibanez J.F. (2021). The Metabolic Features of Tumor-Associated Macrophages: Opportunities for Immunotherapy?. Anal. Cell. Pathol..

[178] Yin H., Lu H., Xiong Y., Ye L., Teng C., Cao X., Li S., Sun S., Liu W., Lv W. (2021). Tumor-Associated Neutrophil Extracellular Traps Regulating Nanocarrier-Enhanced Inhibition of Malignant Tumor Growth and Distant Metastasis. ACS Appl. Mater. Interfaces.

[179] Chaib M., Chauhan S.C., Makowski L. (2020). Friend or Foe? Recent Strategies to Target Myeloid Cells in Cancer. Front. Cell Dev. Biol..

[180] Ringquist R., Ghoshal D., Jain R., Roy K. (2021). Understanding and improving cellular immunotherapies against cancer: From cell-manufacturing to tumor-immune models. Adv. Drug Deliv. Rev..

[181] Yao H., Guo X., Zhou H., Ren J., Li Y., Duan S., Gong X., Du B. (2020). Mild Acid-Responsive “Nanoenzyme Capsule” Remodeling of the Tumor Microenvironment to Increase Tumor Penetration. ACS Appl. Mater. Interfaces.

[182] Liu J., Liao S., Diop-Frimpong B., Chen W., Goel S., Naxerova K., Ancukiewicz M., Boucher Y., Jain R.K., Xu L. (2012). TGF-β blockade improves the distribution and efficacy of therapeutics in breast carcinoma by normalizing the tumor stroma. Proc. Natl. Acad. Sci. USA.

[183] Dolor A., Szoka F.C. (2018). Digesting a Path Forward: The Utility of Collagenase Tumor Treatment for Improved Drug Delivery. Mol. Pharm..

[184] Haque S., Morris J.C. (2017). Transforming growth factor-β: A therapeutic target for cancer. Hum. Vaccines Immunother..

[185] Benson A.B., Wainberg Z.A., Hecht J.R., Vyushkov D., Dong H., Bendell J., Kudrik F. (2017). A Phase II Randomized, Double-Blind, Placebo-Controlled Study of Simtuzumab or Placebo in Combination with Gemcitabine for the First-Line Treatment of Pancreatic Adenocarcinoma. Oncologist.

[186] Hecht J.R., Benson A.B., Vyushkov D., Yang Y., Bendell J., Verma U. (2017). A Phase II, Randomized, Double-Blind, Placebo-Controlled Study of Simtuzumab in Combination with FOLFIRI for the Second-Line Treatment of Metastatic KRAS Mutant Colorectal Adenocarcinoma. Oncologist.

[187] Zhang L., Wang Y., Xia T., Yu Q., Zhang Q., Yang Y., Cun X., Lu L., Gao H., Zhang Z. (2016). Suppression for lung metastasis by depletion of collagen I and lysyl oxidase via losartan assisted with paclitaxel-loaded pH-sensitive liposomes in breast cancer. Drug Deliv..

[188] Koo H., Huh M.S., Sun I.C., Yuk S.H., Choi K., Kim K., Kwon I.C. (2011). In vivo targeted delivery of nanoparticles for theranosis. Acc. Chem. Res..

[189] Twarock S., Reichert C., Bach K., Reiners O., Kretschmer I., Gorski D.J., Gorges K., Grandoch M., Fischer J.W. (2019). Inhibition of the hyaluronan matrix enhances metabolic anticancer therapy by dichloroacetate in vitro and in vivo. Br. J. Pharmacol..

[190] Fridman W.H., Miller I., Sautès-Fridman C., Byrne A.T. (2020). Therapeutic Targeting of the Colorectal Tumor Stroma. Gastroenterology.

[191] Guinney J., Dienstmann R., Wang X., de Reyniès A., Schlicker A., Soneson C., Marisa L., Roepman P., Nyamundanda G., Angelino P. (2015). The consensus molecular subtypes of colorectal cancer. Nat. Med..

[192] Becht E., de Reyniès A., Giraldo N.A., Pilati C., Buttard B., Lacroix L., Selves J., Sautès-Fridman C., Laurent-Puig P., Fridman W.H. (2016). Immune and Stromal Classification of Colorectal Cancer Is Associated with Molecular Subtypes and Relevant for Precision Immunotherapy. Clin. Cancer Res. Off. J. Am. Assoc. Cancer Res..

